# General Dental Practitioners' and Dental Hygienists' Perspectives on the Dutch Guideline for Periodontal Screening, Diagnostics, and Treatment in General Practice

**DOI:** 10.1111/idh.70057

**Published:** 2026-04-08

**Authors:** J. C. L. den Boer, B. A. F. M. van Dam, G. A. van der Weijden, J. J. M. Bruers

**Affiliations:** ^1^ Research and Information Department Royal Dutch Dental Association (KNMT) Utrecht the Netherlands; ^2^ Department of Oral Public Health Academic Center for Dentistry Amsterdam (ACTA), University of Amsterdam and Vrije Universiteit Amsterdam the Netherlands; ^3^ Paro Praktijk Utrecht/Implantologie Utrecht the Netherlands

**Keywords:** dental hygienists, general dental practitioners, guideline, Netherlands, oral healthcare practice, periodontal screening

## Abstract

**Objectives:**

In January 2021, the guideline ‘Periodontal Screening, Diagnosis, and Treatment in General Practice’ was introduced as a successor to the ‘Perio Protocol’. Simultaneously, the screenings tool ‘Dutch Periodontal Screening Index’ (DPSI) was replaced by the ‘Periodic Periodontal Screening’ (PPS). The experiences of general dental practitioners (GDPs) and dental hygienists (DHs) with the new guideline were evaluated in the autumn of 2022, with a focus on the PPS and comparisons to the ‘Perio Protocol.’ The evaluation further aimed to assess how the new guideline contributes to more active involvement of patients in the treatment of periodontal diseases and to identify differences between GDPs and DHs regarding their experiences with the new guideline.

**Methods:**

Random groups of 1000 GDPs and 400 DHs were invited to complete an online questionnaire on this topic: 125 (12.5%) GDPs and 102 (25.5%) DHs responded.

**Results:**

By the end of 2022, a large majority of both GDPs and DHs were using the PPS. Both professional groups indicated that they were satisfied with the new guideline. DHs used the PPS in more cases than GDPs and were also more often satisfied with the new guideline.

**Conclusion:**

The new guideline and screening tool were widely adopted and effectively utilized. Both GDPs and DHs reported satisfaction with the guideline, with DHs being slightly more positive. The guideline's clarity, flexibility, and ease of use were valued, contributing to its successful implementation. Additionally, GDPs and DHs considered the PPS an improvement over the DPSI, particularly in engaging patients in periodontal care.

## 1 Introduction

Based on data from the Netherlands and United States, it can be assumed that 7 to 8% of adults in Western societies suffer from severe periodontal disease [1]. Könönen et al. report a 10% prevalence worldwide, with the severe form mainly increasing between the ages of 30 and 40 [2]. The World Health Organization (WHO) estimates the prevalence to be 18.8% worldwide and 17.7% in prosperous countries [3]. A recent study by Trindade et al. mentions a prevalence of 23.6%, which is higher than former estimates [4]. In a study from the United States, Eke et al. found that over 47% of the population had a mild, moderate, or severe form of periodontal disease [5]. Although the estimates presented above vary due to differences in case definitions and their confidence, it is evident that periodontal disease is a common concern [4]. Associations have been found between periodontal disease and several non‐communicable diseases and systemic disorders [6]. Proper screening, diagnosis, and treatment of periodontal disease seem therefore important for both oral health and the overall health of patients.

### 1.1 Periodontal Protocol and Dutch Periodontal Screening Index

For accurate screening of patients with periodontal diseases, the Dutch Society for Periodontology (NVvP) introduced the Periodontal Diagnosis and Treatment Protocol (the ‘Perio Protocol’), in 1998 [7]. As part of this protocol, the ‘Dutch Periodontal Screening Index’ (DPSI), was introduced, based on the WHO's Community Periodontal Index of Treatment Needs (CPITN) [8]. Initially, the ‘Perio Protocol’ only included a flowchart based on DPSI outcomes, categorizing patients into groups A, B, and C. In 2010, the protocol and use of the DPSI were clarified and emphasized [9].

However, it gradually became apparent that the DPSI allowed for significant differences in the interpretation of probing pocket depth, calculus, bleeding, and recessions, leading to variations in treatment plans [10]. Additionally, the DPSI, intended for screening, was sometimes (mis)used as a diagnostic tool [10]. For patients with higher DPSI scores, a more comprehensive examination of the periodontium is recommended to provide an accurate diagnosis and personalized explanation and treatment advice based on the findings [11].

Because the official ‘Perio protocol’ consisted solely of a flowchart, the NVvP received a warning about its legal risks. The flowchart allowed no deviations or exceptions, raising concerns that it could be seen as a rigid standard in legal proceedings. In response, a committee was formed to draft a new guideline. This new guideline includes a flowchart, detailed clarification, and scientific justification. In January 2021, the guideline, ‘Periodontal Screening, Diagnosis, and Treatment in General Practice,’ replaced the old ‘Perio protocol’. At the same time, the screening tool DPSI was replaced by the ‘Periodic Periodontal Screening’ (PPS) [12]. The latter functions like a traffic light, enabling the oral healthcare provider to make informed decisions based on further findings and considerations. Within this shift, the manual periodontal probe remains the clinical standard for assessing pocket depths and periodontal condition [13]. The inclusion of an additional decision‐making step between screening and treatment (if necessary) improves the new guideline, ensuring a more appropriate and tailored treatment strategy. The new guideline assigns scores of 1, 2, or 3 based on pocket depth, ‘likely adequate’ (green), ‘possibly adequate’ (orange), and ‘probably inadequate’ (red) periodontal condition, respectively (see Supplement 1). Treatment decisions now also consider the patient's needs, wishes and possibilities, involving them in follow‐up treatment choices. The flowchart for periodontal screening, diagnosis and treatment is shown in Supplement 2.

Active involvement of the patient is important, as periodontal health is largely determined by patient's oral hygiene [14]. Oral healthcare providers must therefore pay attention to the patient's attitude, behaviour, and knowledge regarding compliance with self‐care advice [14]. Carra et al. pointed out that the required behavioural change depends on the patient's motivation and willingness to change [15]. Several other authors also emphasize the importance of tailoring the prevention and treatment of periodontitis [10, 16]. Building a relationship of trust with patients is also important [17]. Due to the addition of a decision point and related considerations, the PPS score is not directly linked to treatment, in contrast to the purpose for which the DPSI score was developed and (mis)used in practice. Besides a step‐wise approach to periodontal treatment, the new guideline also outlines the steps for periodontal prevention, which include self‐care and professional prophylaxis guidance.

### 1.2 Role of General Dental Practitioners, Dental Hygienists, and Patients

The introduction of a new guideline is relevant for both general dental practitioners (GDPs) and dental hygienists (DHs), as periodontal treatment falls within the scope of both these professions. However, it cannot be ruled out that these groups may approach and assess the new guideline differently, although Patel et al. found that GDPs and DHs have similar knowledge and approaches to periodontal care [18]. But GDPs perform a wide range of dental treatments, while DHs have more specific tasks related to prevention and periodontal treatment (scaling and root planing), as well as complex prevention, such as subgingival plaque and calculus removal and the treatment of medically compromised patients [19]. It is not surprising, therefore, that within general dental practices, collaboration between GDPs and DHs is common in (periodontal) care [20]. In addition to the oral healthcare providers involved in periodontal treatment, the role of the patient should not be overlooked, especially since behavioural change is generally necessary as well [21]. Therefore, it is important that the guideline is also designed to involve the patient in the treatment process. This was especially true at the time the new guideline was introduced, as the ongoing COVID‐19 pandemic led to a decrease in visits to oral healthcare providers, although in the Netherlands, the decline was comparatively limited [22, 23].

### 1.3 Evaluation

In the autumn of 2022, GDPs and DHs had gained over a year and a half of experience with the new guideline and the PPS. At that time, the NVvP, the Royal Dutch Dental Association (KNMT), and the Dutch Association of Dental Hygienists (NVM) felt the need for an evaluation among both professional groups to determine whether the new guideline was indeed an improvement for them and whether it facilitated patient involvement. Since both GDPs and DHs are involved in periodontal treatments in their own professional capacity, it was important to evaluate the new guideline both in general and separately within each group. The aim of the study was to address this need. The key research questions were: (1) What are the experiences of GDPs and DHs with the use of the new guideline and the PPS, especially in comparison with the ‘Perio Protocol’? (2) To what extent does the new guideline contribute to the desired more active involvement of patients in the treatment of periodontal diseases? (3) Are there differences between GDPs and DHs regarding their experiences with the new guideline?

### 1.4 Study Population and Methodology

The survey was conducted as a cross‐sectional study, using an online questionnaire among a random sample of GDPs and a sample of DHs. The study was previously published in Dutch in the Nederlands Tijdschrift voor Tandheelkunde (NTvT) under the title ‘Tandartsen en mondhygiënisten over de richtlijn Parodontale Screening, Diagnostiek en Behandeling in de algemene praktijk’ [24]. This is a translation, edited for an international audience. The manuscript was developed in consultation with the co‐authors and with permission from the editors‐in‐chief of NTvT.

### 1.5 Research Instrument

For this research project a questionnaire was developed in consultation with the initiators. In order to create valid questions, preliminary semi‐structured interviews were conducted with two GDPs and one DH about their experiences with the new guideline. While no formal validation study was conducted, the questionnaire was developed in collaboration with experts in the field, including individuals involved in the development of the guideline, and in consultation with the NVvP, NVM Mondhygiënisten, and KNMT. Additionally, the KNMT's Research Advisory Committee (Commissie Onderzoeksbegeleiding) reviewed the study design. It contained 16 mainly structured questions with closed answer categories covering topics such as how information about the new guideline was acquired, the determination and recording of the PPS, experiences and overall satisfaction with the new guideline. The full text of the questionnaire is included as Supplement 3. The web form was pre‐tested by the researchers.

### 1.6 Data Collection

In September 2022, an independent research agency (KBA Nijmegen) sent a personal link to an online questionnaire via email to a random sample of 1000 GDPs aged 67 or younger with a residential and/or work address known to the KNMT in the Netherlands. The KNMT Dentist Database includes nearly all GDPs in the Netherlands, and the sample represents the group of GDPs available for dental practice. In October 2022, a similar link was sent to a random sample of 400 DHs made available from the membership base of NVM, which covers about 85% of the active DHs. Both samples represent approximately 10% of each professional group. After two reminders to those who had not yet completed the survey, data collection closed mid‐November 2022. All collected data were subsequently processed by KBA Nijmegen into a pseudonymized data file ensuring that individual GDPs, DHs, and/or practices could not be identified. The researchers received only this coded file for analysis and reporting of the research results.

### 1.7 Data Processing and Analysis

Respondents who answered no questions or only a selection of questions were not included in the analyses. Those who did not complete the questionnaire in full but did answer questions about the new guideline were included. Unexpected combinations of answers were assessed individually and disregarded if necessary.

For the aspects on which PPS and DPSI were compared, it was examined whether there were any interrelated aspects. This was done using Principal Component Analysis with Varimax rotation, where an Eigenvalue of 1 was used to determine the number of factors. This led to two components, which together explained 55.8% of the variance. The first component included the characteristics: easy, clear to patients, clear for healthcare providers, offers freedom, meaningful, promotes patient satisfaction, and indicates the effectiveness of PPS compared to the DPSI. The second component combined the characteristics: labour‐intensive, time‐consuming, and administratively burdensome, indicating the feasibility of PPS compared to DPSI. The details on the rotated component matrix and explained variance are provided in Supplement 4. A sum score was determined for both components, and a reliability analysis was performed. The sum scores for effectiveness and feasibility were calculated as unweighted sums of the respective characteristics within each component. Each characteristic was scored as −1 (preference for DPSI), 0 (no preference), or 1 (preference for PPS). The Cronbach's alpha for the effectiveness component was 0.771, and for the feasibility component, it was 0.777, indicating good internal consistency for both components. The sum score for effectiveness was based on six characteristics and ranged from −6 (very strong preference for DPSI) to 6 (very strong preference for PPS). The feasibility score was based on three characteristics and ranged from −3 (very strong preference for DPSI) to 3 (very strong preference for PPS). Two of the eleven characteristics were not taken into account: sufficiently reimbursed and leads to high costs for the patient, because more than four‐fifths of both GDPs and DHs (85.7% or higher) indicated that these aspects applied equally to both PPS and DPSI.

In all analyses, comparisons were made between the groups of GDPs and DHs using the Chi‐squared test or the independent *t*‐test. Because the ratio of the number of GDPs to DHs in the study was not in line with the numerical ratios of both total populations, the results for the entire research group are not stated. Correlations were considered statistically significant if *p* < 0.05. All calculations and analyses were performed using SPSS for Windows (version 28).

### 1.8 Ethics Statement

The invitation to participate in the study informed GDPs and DHs that their involvement was voluntary. By submitting their completed questionnaire, participants gave consent for their information to be used for the study. The use of data for additional (secondary) analyses pertaining to this study was approved by the Institutional Review Board of the Academic Center for Dentistry Amsterdam (ACTA)—ACTA‐ETC—under application number 2024–96888.

## 2 Results

### 2.1 Response

Ultimately, 227 oral healthcare providers (125 GDPs and 102 DHs) completed the questionnaire. The total response rate was 16.2%, with 12.5% among GDPs and 25.5% among DHs, resulting in a relative underrepresentation of GDPs. The questionnaire did not include questions about the background characteristics of the respondents. Unfortunately, this made it impossible to perform analyses to determine the extent to which the group of DHs was representative of the entire population. However, as KBA Nijmegen and the KNMT structurally collaborate in carrying out research, for GDPs some background information could be linked to the data based on the respondent number. Considering gender, age, place of graduation, and region of establishment, it was determined that the GDPs in the research group were reasonably representative of the entire profession. In our sample, 52% of the respondents were female versus 48% in the population, with a mean age of 44.4 years, compared to 45.5. Regarding place of graduation, 44% had graduated in Amsterdam, 16% in Groningen, 19% in Nijmegen, 2% in Utrecht, and 19% outside the Netherlands, compared to 39%, 13%, 21%, 6%, and 21%, respectively. Additionally, 13% of the respondents were established in the north, 15% in the east, 21% in the south, and 51% in the west, compared to 10%, 17%, 19%, and 54%.

### 2.2 Familiarity With the New Guideline

All respondents indicated they had become aware of the new guideline primarily through self‐study (63.6% of GDPs and 75.0% of DHs, respectively), professional journals (60.8% and 58.3%), and/or information provided by the NVvP via a webinar (25.8% and 54.8%). Fewer mentions were made of the NVvP videos on YouTube (9.3% and 10.7%) and the NVvP conference meetings about the guideline (7.2% and 13.1%).

### 2.3 Determining and Recording PPS

Regarding PPS, nearly six in ten GDPs (59.8%) and DHs (57.4%) consistently screened their patients according to the PPS during a periodic oral examination. Among GDPs, 24.6% usually did this, 9.0% sometimes, and 6.6% never did. For DHs, these percentages were 34.7%, 4.9%, and 3.0%, respectively. On average, GDPs determined the PPS in 90.0% of their adult patients (18 years or older) and 77.5% of their young patients (17 years or younger), while DHs determined it in 93.2% and 78.8%, respectively. GDPs spent less time determining and recording the PPS compared to DHs (2.3 versus 4.2 min, t = −3.946, df = 116; *p* < 0.001). Regarding the frequency of recording the PPS in the patient file, 86.7% of GDPs consistently recorded it, 11.4% did so occasionally, and 1.9% never recorded it. Among DHs, these figures were 78.7%, 20.2%, and 1.1%, respectively. Table 1 provides an overview of the data recorded by GDPs and DHs.

**TABLE 1:** Data that general dental practitioners and dental hygienists recorded in the patient file after determining the Periodic Periodontal Screening score (PPS).

	General dental practitioners	Dental hygienists
PPS‐scores of measurements per quadrant	81%	81%
General oral hygiene of the patient^a^	76%	88%
Oral information/instruction provided	70%	81%
Possible referrals	66%	55%
Follow‐up appointments^b^	63%	77%
Presence of calculus	55%	58%
General PPS‐score (a, b or c), or	53%	59%
Location of calculus	42%	40%
Patient motivation^c^	33%	60%
Written information/instruction provided (brochures, etc.)^d^	18%	58%
Other data^b^	7%	17%
N	100	88

*Note:* Multiple responses allowed.

^a^ Chi^2^ = 4086; df = 1; *p* = 0,043.

^b^ Chi^2^ = 4513; df = 1; *p* = 0,034.

^c^ Chi^2^ = 13,981; df = 1; *p* < 0,001.

^d^ Chi^2^ = 32,165; df = 1; *p* < 0,001.

### 2.4 Use of Educational Materials

Fewer GDPs than DHs indicated that they used specially developed educational materials to inform patients about the periodontium and periodontal treatment options (64.9% versus 89.3%, Chi^2^ = 14.721, df = 1, *p* < 0.001). Among these GDPs, 52.4% worked with information leaflets made available from the NVvP, while approximately three‐quarters (76.0%) of DHs did so (Chi^2^ = 8.420, df = 1, *p* = 0.004). Additionally, 49.2% of GDPs and 58.7% of DHs used ’instruction cards’ also made available from the NVvP. Furthermore, 14.3% of GDPs and 5.3% of DHs used information from the NVvP website, and 39.7% of GDPs and 25.3% of DHs used other information materials as well.

### 2.5 Assessment of Effectiveness and Feasibility of PPS

For this evaluation, GDPs and DHs compared the PPS with the DPSI on various aspects. A complete overview of all features can be found in Supplement 5. Table 2 presents the scores for the ‘effectiveness’ and ‘feasibility’ scales, based on their assessments. Half of GDPs (50.0%) and a majority of DHs (60.7%) indicated a preference for the PPS in combination with the new guideline in terms of effectiveness. Regarding feasibility, more than half of both GDPs and DHs (53.0% and 58.3%, respectively) had no preference between PPS and DPSI. However, the proportion preferring PPS was larger in both groups compared to those preferring DPSI, with 39.8% of GDPs preferring PPS versus 7.2% preferring DPSI, and 35.7% of DHs preferring PPS versus 6.0% preferring DPSI. Statistically, there was no significant difference between GDPs and DHs in their preferences for both effectiveness and feasibility comparisons.

**TABLE 2:** Attitudes of general dental practitioners (GDPs) and dental hygienists (DHs) towards the effectiveness and feasibility of the Periodic Periodontal Screening (PPS) compared to the Dutch Periodontal Screening Index (DPSI).

	Effectiveness		Feasibility	
	GDPs	DHs	GDPs	DHs
Strongly preferred DPSI	5.1%	4.8%	3.1%	4.8%
Preferred DPSI	23.5%	25.0%	4.1%	1.2%
No preference DPSI or PPS	23.4%	9.5%	53.0%	58.3%
Preferred PPS	33.7%	40.5%	8.2%	9.5%
Strongly preferred PPS	14.3%	20.2%	31.6%	26.2%
Mean	0.55	1.08	0.73	0,68
Median	0	1	0	0
Standard deviation	2.41	2.78	1.26	1.41
Minimum	−6	−5	−2	−3
Maximum	6	6	3	3
*n*	98	84	98	84
Cronbach's alpha	0,771		0,777	

### 2.6 Involving Patients

Respondents were presented with eight statements concerning the communication and involvement of patients in periodontal treatment. Table 3 shows that the majority of GDPs (43.6%) and the majority of DHs (75.3%) ‘strongly agreed’ or ‘agreed’ with the statement that the guideline offers more options to consider the patient's wishes and circumstances compared to the ‘Perio Protocol’. Similarly, a majority of both GDPs (42.6%) and DHs (64.2%) agreed with the statement that the guideline provides more opportunities to collaboratively develop a treatment approach with the patient. Additionally, 40.6% of GDPs and 58.1% of DHs agreed that the guideline offers many tools to inform patients about periodontal problems and their treatment. Finally, 46.8% of GDPs and 51.9% of DHs expressed that additional training on ‘motivational interviewing’ would benefit them or their practice staff.

**TABLE 3:** Extent to which general dental practitioners (GDPs) and dental hygienists (DHs) disagree or agree with statements about the possibilities that the guideline ‘Periodontal Screening, Diagnostics, and Treatment in General Practice’ offers to involve patients in and inform them about the treatment.

	strongly disagree	somewhat disagree	neither disagree nor agree	somewhat agree	strongly agree	don't know
Compared to the ‘periodontal protocol’, the guideline offers more options for developing a treatment approach together with the patient komen^a^						
GDPs		10.6%	40.4%	30.9%	11.7%	6.4%
DHs	3.7%	2.5%	28.4%	32.1%	32.1%	1.2%
Compared to the ‘periodontal protocol’, the guideline offers more options for taking the patient's wishes and circumstances into account in a treatment plan^b^						
GDPs	2.1%	9.6%	41.5%	30.8%	12.8%	3.2%
DHs		5,0%	18.5%	42.0%	33.3%	1.2%
In response to the guideline, I have informed patients more extensively than before about a referral for periodontal treatment						
GDPs	22.3%	19.1%	41.5%	8.5%	4.3%	4.3%
DHs	17.3%	12.3%	53.1%	13.6%	3.7%	
The guideline offers many tools to inform the patient about periodontal problems and their treatment^c^						
GDPs	2.1%	11.7%	41.5%	27.7%	12.8%	4.2%
DHs		1.2%	37.0%	42.0%	16.1%	3.7%
In response to the guideline, I have started to take more account of the oral healthcare goals of the patients in question when determining a periodontal treatment plan						
GDPs	20.2%	17.0%	42.6%	11.7%	6.4%	2.1%
DHs	19.7%	17.3%	42.0%	18.5%	2.5%	
The guideline ensures that patients have more say in treatment decisions^d^						
GDPs	13.8%	13.8%	43.6%	16.0%	6.4%	6.4%
DHs	3.7%	6.2%	38.3%	27.1%	19.5%	6.2%
The guideline has encouraged me to use the techniques of ‘motivational interviewing’ in information and instruction						
GDPs	11.7%	13.8%	51.1%	16.0%	3.2%	4.2%
DHs	7.4%	13.6%	39.5%	28.4%	7.4%	3.7%
Additional training on ‘motivational interviewing’ would be welcome for me and/or the employees in the practice						
GDPs	4.3%	12.8%	34.0%	33.0%	13.8%	2.1%
DHs	6.2%	14.8%	25.9%	30.9%	21.0%	1.2%

*Note: n* = 94 (GDPs)/81 (DHs).

^a^ Chi^2^ = 20,988; df = 5; *p* < 0,001.

^b^ Chi^2^ = 20,905; df = 5; *p* < 0,001.

^c^ Chi^2^ = 11,857; df = 5; *p* = 0,037.

^d^ Chi^2^ = 15,587; df = 5; *p* = 0,008.

### 2.7 General Satisfaction

Table 4 illustrates that almost half (46.2%) of GDPs were (very) satisfied with the replacement of the former ‘Perio Protocol’ by the new guideline, while 35.5% were neutral and 18.3% were dissatisfied. Among DHs, 73.5% expressed to be (very) satisfied, 18.1% were neutral, and 8.4% were (very) dissatisfied. Regarding the perceived opportunities provided by the guideline, 34.1% of GDPs and 27.7% of DHs agreed that it offers more opportunities for GDPs in general practice to fulfil their pivotal role in oral healthcare provision, with 12.8% of both GDPs and DHs being neutral, and 9.9% disagreeing. Additionally, 48.9% of GDPs and 61.7% of DHs agreed that the guideline presents them with more opportunities to demonstrate their value in the field of periodontal treatment, with 9.6% of GDPs and 7.4% of DHs disagreeing with this statement.

**TABLE 4:** The extent to which general dental practitioners and dental hygienists are satisfied or dissatisfied with the fact that the paro protocol has been replaced by the new guideline believes that the new guideline.

	General dental practitioners	Dental hygienists
Very satisfied	4.3%	12.1%
Satisfied	41.9%	61.4%
Neither satisfied nor dissatisfied	35.5%	18.1%
Dissatisfied	16.1%	7.2%
Very dissatisfied	2.2%	1.2%
*n*	93	83

*Note:* Chi^2^ = 14.594; df = 4; *p* = 0,006.

## 3 Discussion

This study shows that the vast majority of both GDPs and DHs always or usually screen their patients and record their measurements according to the PPS. The use of the former screening tool, the DPSI, was assessed in 1998 and 2002, with surveys limited to GDPs only [25]. At that time, fewer GDPs used the DPSI as a standard measurement for screening. However, it is unclear to what extent the adoption of the new screening tool PPS has influenced this increase, given numerous developments in recent years that could also have promoted periodontal screening [20]. More recent research from Belgium also indicates the less frequent use of the DPSI [26]. Additionally, in 1998 and 2002, GDPs spent more time measuring the DPSI in patients compared to measuring the PPS in 2022. It is unknown whether the activities on which the time is spent differ between the former ‘Perio Protocol’ and the new guideline, as the questionnaire did not provide sufficient information to explore this. A qualitative study may be more suitable for investigating this aspect.

Furthermore, satisfaction among GDPs with the new guideline and the PPS appears to be somewhat greater than with the former ‘Perio Protocol’ and DPSI during evaluations conducted a few years after its introduction [25]. The DPSI was considered valuable for screening the periodontal status during periodic oral examinations [7]. However, the PPS is seen as more user‐friendly. It is also notable that GDPs and DHs often searched for further information about the guideline on their own, perhaps partly due to the limited opportunities to learn about it through other means during the COVID‐19 pandemic. This independent search for information suggests that the guideline aligns with the organizational culture in oral healthcare practices in the Netherlands [27]. Perhaps the old ‘Perio Protocol’, which had been in use for a long time, played a preparatory role in this.

Regarding the extent to which the new guideline contributes to more active patient involvement in periodontal treatment, both GDPs and DHs are largely positive. In other words, the new guideline facilitates adapting treatment to the patient's knowledge, motivation, and behaviour, which are critical factors for the success of periodontal treatment [14, 15, 21].

There was no significant difference between GDPs and DHs in their adoption of the new guideline. This is consistent with findings from research from the United States indicating similar approaches in periodontal therapy among GDPs and DHs, suggesting that both groups utilize comparable methods [18]. However, differences in practice are evident. DHs tend to more frequently record certain aspects and dedicate more time, on average, to measuring and recording the PPS score compared to GDPs. This disparity may be attributed to the greater emphasis that DHs place on periodontal treatment in their daily practice compared to GDPs, as well as differences in training, professional focus, and role distribution within dental practices [19, 20, 28].

## 4 Strengths and Limitations

One of the strengths of this study is its inclusion of both GDPs and DHs, making it more representative of Dutch oral health care providers compared to previous evaluations of the DPSI, which only surveyed GDPs [25]. However, some considerations should be noted regarding survey response rates. Initially, 26% of invited DHs participated, whereas only 13% of GDPs did so. According to Rowly, a response rate of 20% is generally considered acceptable, a threshold not met in this study for GDPs [29]. Overall, there has been a decline in GDPs' willingness to participate in surveys, which may have contributed to non‐response bias [30]. Consequently, it could be argued that the differences observed between GDPs and DHs might therefore be more or less pronounced than reflected in this study. Future research could also consider including prophylaxis assistants, another group of oral healthcare providers active in general dental practices in the Netherlands, in the research group.

Another point of concern is the composition of the study's participant groups. Based on registration data indicating 9376 active GDPs as of January 2023 and an estimated 3600 active DHs in 2019, the total ratio of GDPs to DHs is approximately 72% to 28% [31]. However, in this research group, the ratio was 55% GDPs to 45% DHs, indicating a relative underrepresentation of GDPs. As a result, only separate results for GDPs and DHs are presented, and not results for the entire combined group.

Another limitation was the availability of background data. For DHs, this data was limited at the population, sample, and respondent levels. This made it impossible to determine whether the group of participants was representative of the DH population in the Netherlands. The participating GDPs can be considered a reasonable reflection of the population. Since the circumstances for both groups were comparable, there is no reason to assume that the situation for DHs was fundamentally different.

In developing the questionnaire, two steps were taken to promote its validity. First, based on targeted semi‐structured interviews with GDPs, an image was obtained of what users were experiencing with regard to the guideline. Furthermore, the questionnaire was developed in close collaboration with the NVvP, in which periodontology‐oriented GDPs and DHs are united. However, no formal validation was conducted, which should be acknowledged as a limitation of this study. Another limitation concerns the lack of formal validation of the questionnaire. On the other hand, the research design and questionnaire were carefully developed in collaboration with experts from the field and in consultation with the relevant scientific and professional associations.

## 5 Implications for Practice

A year and a half after the introduction of the new guideline, it appeared to be well implemented, with both GDPs and DHs using it extensively. Not only did GDPs and DHs see benefits for themselves but they also recognized the advantages for the patients. The better assessment of the screening instrument PPS compared to the DPSI in terms of effectiveness and feasibility likely contributed significantly to this. The new guideline has been implemented for several years now. After this period, it is essential to assess whether the guideline still aligns with current scientific knowledge. Should this evaluation lead to adjustments or revisions, it is important to consider the differences between GDPs and DHs. Additionally, ensuring sufficient opportunities for continuing education and professional development for both groups is essential to support the effective implementation of any updates. Any modifications should accommodate the needs of both groups without compromising usability. Ultimately, a guideline that supports effective practice for both GDPs and DHs will maximize benefits for patients.

## 6 Conclusion

More than a year and a half after their introduction, the new guideline and the screening tool PPS were widely adopted and effectively utilized. Efforts by organizations such as the NVvP, KNMT, and NVM played a key role in preparing professionals for these changes. Both GDPs and DHs reported satisfaction with the guideline, with DHs being slightly more positive. The guideline's clarity, flexibility, and ease of use were valued, contributing to its successful implementation. Additionally, GDPs and DHs considered the PPS an improvement over the DPSI, particularly in engaging patients in periodontal care.

## 7 Clinical Relevance

*Scientific rationale for the study:* Dutch general dental practitioners (GDPs) and dental hygienists (DHs) are each affected by the new guideline on periodontal screening, diagnosis, and treatment in general practice, though from different perspectives. This evaluation takes both perspectives into account. *Principal findings:* The new guideline and its accompanying screening tool are well known and widely used by both GDPs and DHs. Compared to its predecessor, the guideline is easier to use and offers greater flexibility. *Practical implications:* The new guideline provides better opportunities for GDPs and DHs to engage patients in their periodontal care.

## Author Contributions

J.C.L.B. and JB developed the questionnaire and led the data collection, J.C.L.B. and BVD analysed the data and made the original draft, which was edited by GVDW and JB, J.C.L.B. and GVDW led the translation from Dutch.

## Funding

The research was carried out within the framework of the Data Stations project, which is funded by the Royal Dutch Dental Association (KNMT).

## Conflicts of Interest

The authors declare no conflicts of interest.

## Supporting information


**Supplement 1:** A summary of considerations for the use at the decision point following a PPS score of 1, 2, or 3.
(GDPsAndDHsOnNewDutchGuidelinePeriondontologyInGeneralPractice_IDH_Supplement 1.docx).

**Supplement 2:** Flowchart for periodontal screening, diagnosis and treatment in general practice, as included in the new guideline.
(GDPsAndDHsOnNewDutchGuidelinePeriondontologyInGeneralPractice_IDH_Supplement 2.docx).

**Supplement 3:** Questionnaire for the evaluation of the Dutch guideline “Periodontal Screening, Diagnosis and Treatment in General Practice”.
(GDPsAndDHsOnNewDutchGuidelinePeriondontologyInGeneralPractice_IDH_Supplement 3.docx).

**Supplement 4:** Rotated component matrix with eigenvalues and explained variance (GDPsAndDHsOnNewDutchGuidelinePeriondontologyInGeneralPractice_IDH_Supplement 4.docx).

**Supplement 5:** Opinion of general dental practitioners and dental hygienists on whether certain characteristics apply more or less to the PPS compared to the DPSI.
(GDPsAndDHsOnNewDutchGuidelinePeriondontologyInGeneralPractice_IDH_Supplement 5.docx).

## Data Availability

The data that support the findings of this study are available from the corresponding author upon reasonable request.
