# Supplementary material for: General Dental Practitioners' and Dental Hygienists' Perspectives on the Dutch Guideline for Periodontal Screening, Diagnostics, and Treatment in General Practice
Source: Int J Dent Hyg. 2026 Apr 8;24(4):711–9. doi: 10.1111/idh.70057 (PMC13622924; doi:10.1111/idh.70057)
Supplement: Supplementary file 1 [file IDH-24-711-s001.docx]

**Supplement 2.	Flowchart for periodontal screening, diagnosis and treatment in general practice, as included in the new guideline**
