# Supplementary material for: General Dental Practitioners' and Dental Hygienists' Perspectives on the Dutch Guideline for Periodontal Screening, Diagnostics, and Treatment in General Practice
Source: Int J Dent Hyg. 2026 Apr 8;24(4):711–9. doi: 10.1111/idh.70057 (PMC13622924; doi:10.1111/idh.70057)
Supplement: Supplementary file 2 [file IDH-24-711-s002.docx]

**Supplement 3.	Questionnaire for the evaluation of the Dutch guideline “Periodontal Screening, Diagnosis and Treatment in General Practice”**

*The guideline 'Periodontal Screening, Diagnostics and Treatment in general practice' was published in January 2021. This guideline replaces the ’Perio Protocol’, introduced in 1998. The 'Dutch Periodontal Screenings Index' (DPSI) has simultaneously been replaced as a screening index by the 'Periodic Periodontal Screening' (PPS).*

| 0 | Are you a general dental practitioner or dental hygienist working in patient treatment? | | |
| --- | --- | --- | --- |
|  | 1 | yes | ***--> to question 1*** |
|  | 2 | no | ***--> to end*** |

| 1 | Do you determine the PPS during the routine oral examinations of your patients? | | |
| --- | --- | --- | --- |
|  | 1 | yes, always | ***--> to question 5*** |
|  | 2 | yes, usually | ***--> to question 3*** |
|  | 3 | yes, sometimes | ***--> to question 3*** |
|  | 4 | no, never | ***--> to question 2*** |

| 2 | For what reason(s) do you never determine the PPS during a routine oral examination?  *(multiple responses allowed)* | |
| --- | --- | --- |
|  | 1 | patients are not motivated |
|  | 2 | the index is unmanageable |
|  | 3 | I use a different screening index (for example, DPSI) |
|  | 4 | it takes too much (extra) time |
|  | 5 | the standard is set too high |
|  | 6 | this index is too inflexible, too rigid |
|  | 7 | the reward for using the new guideline is insufficient |
|  | 8 | someone else in the practice determines the PPS |
|  | 9 | other reason, namely: |
|  |  | ***--> to end*** |

| 3 | For what reason(s) do you not always determine the PPS during a routine oral examination?  *(multiple responses allowed)* | |
| --- | --- | --- |
|  | 1 | certain patients are not motivated. |
|  | 2 | it takes me too much (extra) time. |
|  | 3 | I only do it with certain age groups. |
|  | 4 | certain patients have already been screened and referred to a dental hygienist/periodontist. |
|  | 5 | it is not necessary for patients with (very) good oral health. |
|  | 6 | the remuneration for using this screening instrument is insufficient. |
|  | 7 | other reason, namely: |

| 4 | Can you indicate approximately what percentage of your adult and juvenile patients you determine a PPS during a routine oral examination? | |
| --- | --- | --- |
| a | In adult patients (18 years or older) | . . . . . % *(enter percentage)* |
| b | In young patients (17 years or younger) | . . . . . % *(enter percentage)* |

| 5 | On average, how many minutes do you spend determining (and recording) the PPS per patient? |
| --- | --- |
|  | . . . . . minutes *(enter number)* |

| 6 | Do you record the PPS score in the patient file once you have determined it? | | |
| --- | --- | --- | --- |
|  | 1 | yes, always | ***--> to question 8*** |
|  | 2 | yes, usually |  |
|  | 3 | yes, sometimes |  |
|  | 4 | no, never | ***--> to question 9*** |

| 7 | In which cases do you record the PPS score?  *(multiple responses allowed)* | |
| --- | --- | --- |
|  | 1 | in new patients |
|  | 2 | in case of a PPS score of 2 or higher |
|  | 3 | in case of a PPS score of 3 |
|  | 4 | if the PPS score has changed compared to the previous measurement |
|  | 5 | in adult patients (18 years or older) |
|  | 6 | in young patients (17 years or younger) |
|  | 7 | in patients with an increased risk of periodontal problems |
|  | 8 | in other cases, namely: |

| 8 | Which data do you record in the administration after determining a PPS for a patient?  *(multiple responses allowed)* | |
| --- | --- | --- |
|  | 1 | PPS scores of measurements per quadrant |
|  | 2 | general PPS score (1, 2 or 3) |
|  | 3 | presence of calculus |
|  | 4 | location of calculus |
|  | 5 | general oral hygiene of the patient |
|  | 6 | patient motivation |
|  | 7 | oral information/instruction provided |
|  | 8 | written information/instruction provided (brochures, etc.) |
|  | 9 | follow-up appointments |
|  | 10 | possible refferals |
|  | 11 | other data, namely: |

| 9 | Below are a number of characteristics related to screening the periodontium. Can you indicate for each characteristic whether it applies more, equally, or less to the PPS compared to the DPSI? | | | |
| --- | --- | --- | --- | --- |
|  |  | *applies* ***more*** *to PPS than to DPSI* | *applies* ***equally*** *to PPS and DPSI* | *applies* ***less*** *to PPS than to DPSI* |
| a | easy |  |  |  |
| b | meaningful |  |  |  |
| c | labour intensive |  |  |  |
| d | time consuming |  |  |  |
| e | sufficiently compensated |  |  |  |
| f | administratively burdensome |  |  |  |
| g | clear for healthcare provider |  |  |  |
| h | clear to patient |  |  |  |
| i | offers freedom |  |  |  |
| j | promotes patient satisfaction |  |  |  |
| k | leads to high costs for the patient |  |  |  |

| 10 | Overall, how satisfied or dissatisfied are you with the replacement of the 'Paro protocol' with the guideline *'Periodontal Screening, Diagnosis, and Treatment in General Practice'*? | |
| --- | --- | --- |
|  | 1 | very satisfied |
|  | 2 | satisfied |
|  | 3 | neither satisfied nor dissatisfied |
|  | 4 | dissatisfied |
|  | 5 | very dissatisfied |
|  | 6 | don’t know |

| 11 | Do you use specially developed educational materials when educating patients about the periodontium and periodontal treatments? | | |
| --- | --- | --- | --- |
|  | 1 | yes |  |
|  | 2 | no | ***--> to question 13*** |

| 12 | What educational tools do you use to educate patients about the periodontium and periodontal treatments?  *(multiple responses allowed)* | | |
| --- | --- | --- | --- |
|  | 1 | educational leaflets from the NVvP |  |
|  | 2 | the plasticized 'instruction cards' of the NVvP |  |
|  | 3 | information on the NVvP website |  |
|  | 4 | other educational material, namely: |  |

| 13 | How did you inform yourself about the guideline *'Periodontal Screening, Diagnostics, and Treatment in General Practice'*?  *(multiple responses allowed)* | | |
| --- | --- | --- | --- |
|  | 1 | I have studied the guideline myself |  |
|  | 2 | I have read information about the guideline in professional journals |  |
|  | 3 | I participated in an NVvP webinar about the guideline |  |
|  | 4 | watched NVvP videos about the guideline on YouTube |  |
|  | 5 | I attended an NVvP meeting about the guideline |  |
|  | 6 | another way, namely: |  |

| 14 | Below are a number of statements about the guideline *'Periodontal Screening, Diagnostics, and Treatment in General Practice.'* Could you please indicate whether you agree or disagree with each statement? | | | | | |
| --- | --- | --- | --- | --- | --- | --- |
|  | *strongly*  *disagree* | *somewhat*  *disagree* | *neither*  *disagree, nor agree* | *somewhat agree* | *strongly agree* | *don’t know* |
| a | Compared to the 'periodontal protocol', the guideline offers more options for developing a treatment approach together with the patient. | | | | | |
|  | 1 | 2 | 3 | 4 | 5 | 6 |
| b | Compared to the 'periodontal protocol', the guideline offers more options for taking the patient's wishes and circumstances into account in a treatment plan. | | | | | |
|  | 1 | 2 | 3 | 4 | 5 | 6 |
| c | In response to the guideline, I have informed patients more extensively than before about a referral for periodontal treatment. | | | | | |
|  | 1 | 2 | 3 | 4 | 5 | 6 |
| d | The guideline offers many tools to inform the patient about periodontal problems and their treatment. | | | | | |
|  | 1 | 2 | 3 | 4 | 5 | 6 |
| e | In response to the guideline, I have started to take more account of the oral healthcare goals of the patients in question when determining a periodontal treatment plan. | | | | | |
|  | 1 | 2 | 3 | 4 | 5 | 6 |
| f | The guideline ensures that patients have more say in treatment decisions. | | | | | |
|  | 1 | 2 | 3 | 4 | 5 | 6 |
| g | The guideline has encouraged me to use the techniques of 'motivational interviewing' in information and instruction. | | | | | |
|  | 1 | 2 | 3 | 4 | 5 | 6 |
| h | Additional training on 'motivational interviewing' would be welcome for me and/or the employees in the practice. | | | | | |
|  | 1 | 2 | 3 | 4 | 5 | 6 |
| i | The guideline offers general dental practitioners in general practice more opportunities to fulfil their pivotal role in providing dental care. | | | | | |
|  | 1 | 2 | 3 | 4 | 5 | 6 |
| j | The guideline offers dental hygienists more opportunities to distinguish themselves in the field of periodontal treatment. | | | | | |
|  | 1 | 2 | 3 | 4 | 5 | 6 |

| 15 | If you have suggestions to improve the guideline 'Periodontal Screening, Diagnosis, and Treatment in General Practice,' you can post them below. |
| --- | --- |
|  |  |

*Thank you for your cooperation.*
