# Supplementary material for: General Dental Practitioners' and Dental Hygienists' Perspectives on the Dutch Guideline for Periodontal Screening, Diagnostics, and Treatment in General Practice
Source: Int J Dent Hyg. 2026 Apr 8;24(4):711–9. doi: 10.1111/idh.70057 (PMC13622924; doi:10.1111/idh.70057)
Supplement: Supplementary file 3 [file IDH-24-711-s003.docx]

**Supplement 4.**	**Rotated component matrix with eigenvalues and explained variance**

|  | *factor 1: effectiveness* | *factor 2: feasibility* |
| --- | --- | --- |
| easy | 0.507 | 0.305 |
| meaningful | 0.772 | -0.050 |
| not labour intensive | -0.067 | 0.862 |
| not time consuming | 0.051 | 0.900 |
| not administratively burdensome | 0.155 | 0.694 |
| clear for healthcare provider | 0.798 | -0.087 |
| clear to patient | 0.757 | 0.108 |
| offers freedom | 0.664 | -0.005 |
| promotes patient satisfaction | 0.554 | 0.177 |
| Eigenvalue | 2.970 | 2.054 |
| Explained variance (%) | 33.0% | 22.8% |
