# Supplementary material for: General Dental Practitioners' and Dental Hygienists' Perspectives on the Dutch Guideline for Periodontal Screening, Diagnostics, and Treatment in General Practice
Source: Int J Dent Hyg. 2026 Apr 8;24(4):711–9. doi: 10.1111/idh.70057 (PMC13622924; doi:10.1111/idh.70057)
Supplement: Supplementary file 5 [file IDH-24-711-s005.docx]

**Supplement 5.	Opinion of general dental practitioners and dental hygienists on whether certain characteristics apply more or less to the PPS compared to the DPSI**

|  | general dental practitioners | | | dental hygienists | | |
| --- | --- | --- | --- | --- | --- | --- |
|  | applies to PPS compared to DPSI | | | applies to PPS compared to DPSI | | |
|  | more | equally | less | more | equally | less |
| easy | 57,1% | 33,7% | 9,2% | 45,2% | 47,6% | 7,2% |
| clear to patient | 30,6% | 55,1% | 14,3% | 40,5% | 45,2% | 14,3% |
| clear for healthcare provider | 24,5% | 40,8% | 34,7% | 29,7% | 40,5% | 29,8% |
| offers freedom ^*^ | 22,4% | 64,3% | 13,3% | 42,9% | 47,6% | 9,5% |
| meaningful | 15,3% | 58,2% | 26,5% | 19,1% | 61,9% | 19,0% |
| promotes patient satisfaction | 7,1% | 88,8% | 4,1% | 15,5% | 79,7% | 4,8% |
| labour intensive | 4,1% | 63,3% | 32,6% | 9,5% | 61,9% | 28,6% |
| time consuming | 4,1% | 64,3% | 31,6% | 4,8% | 64,3% | 30,9% |
| administratively burdensome | 3,1% | 76,5% | 20,4% | 5,9% | 65,5% | 28,6% |
| leads to high costs for the patient | 2,0% | 89,8% | 8,2% | 4,8% | 85,7% | 9,5% |
| sufficiently compensated | 2,0% | 93,9% | 4,1% | 3,6% | 88,1% | 8,3% |
| n | 98 | | | 84 | | |
| * Chi^2^ = 8,680; df = 2; p = 0,013 | | | | | | |
